# Different inocula produce distinctive microbial consortia with similar lignocellulose degradation capacity

**DOI:** 10.1007/s00253-016-7516-6

**Published:** 2016-05-12

**Authors:** Larisa Cortes-Tolalpa, Diego Javier Jiménez, Maria Julia de Lima Brossi, Joana Falcão Salles, Jan Dirk van Elsas

**Affiliations:** Genomics Research in Ecology and Evolution in Nature, Groningen Institute for Evolutionary Life Sciences, University of Groningen, Nijenborgh 7, 9747 AG Groningen, The Netherlands

**Keywords:** Lignocellulose, Degrader communities, Bacterial–fungal consortia, (Hemi) cellulolytic activity, Recalcitrant substrate

## Abstract

Despite multiple research efforts, the current strategies for exploitation of lignocellulosic plant matter are still far from optimal, being hampered mostly by the difficulty of degrading the recalcitrant parts. An interesting approach is to use lignocellulose-degrading microbial communities by using different environmental sources of microbial inocula. However, it remains unclear whether the inoculum source matters for the degradation process. Here, we addressed this question by verifying the lignocellulose degradation potential of wheat (*Triticum aestivum*) straw by microbial consortia generated from three different microbial inoculum sources, i.e., forest soil, canal sediment and decaying wood. We selected these consortia through ten sequential-batch enrichments by dilution–to–stimulation using wheat straw as the sole carbon source. We monitored the changes in microbial composition and abundance, as well as their associated degradation capacity and enzymatic activities. Overall, the microbial consortia developed well on the substrate, with progressively-decreasing net average generation times. Each final consortium encompassed bacterial/fungal communities that were distinct in composition but functionally similar, as they all revealed high substrate degradation activities. However, we did find significant differences in the metabolic diversities per consortium: in wood-derived consortia cellobiohydrolases prevailed, in soil-derived ones β-glucosidases, and in sediment-derived ones several activities. Isolates recovered from the consortia showed considerable metabolic diversities across the consortia. This confirmed that, although the overall lignocellulose degradation was similar, each consortium had a unique enzyme activity pattern. Clearly, inoculum source was the key determinant of the composition of the final microbial degrader consortia, yet with varying enzyme activities. Hence, in accord with Beyerinck’s, “everything is everywhere, the environment selects” the source determines consortium composition.

**Electronic supplementary material:**

The online version of this article (doi:10.1007/s00253-016-7516-6) contains supplementary material, which is available to authorized users.

## Introduction

Lignocellulosic substrates such as wheat, grass and maize straws constitute important carbon and energy sources for microorganisms. In addition to diverse small molecules, cellulose and hemicellulose, both of which can be hydrolyzed to sugars for further biological or chemical utilization (Xu et al. 2013), and lignin constitute the major carbonaceous components in these substrates. Whereas high-value products such as biofuels and diverse building blocks for industrial products can be produced on the basis of the released compounds, the lignin moiety—a polymer of aromatic compounds like phenol—constitutes an important source material for the industrial production of adhesive resin and lignin gels. In plant tissue, the three moieties form a complex structure with intricate bonds, part of which is very difficult to breakdown. Thus, despite multiple research efforts, the current strategies for exploitation of lignocellulosic plant matter are still far from optimal, being hampered mostly by the challenge of degrading the recalcitrant parts of all three moieties.

In natural systems, lignocellulose degradation is carried out by multiple—coexisting—lignocellulolytic microorganisms. These include a range of fungi and bacteria capable of producing a variety of degrading enzymes. These microorganisms most likely establish synergistic relationships among each other and/or with other, non-cellulolytic, microbial species and these interactions are expected to enhance the substrate degradation rates (Lynd et al. 2002). For instance, in forest soils, fungal and bacterial communities play important roles; the former explore dead plant matter even at low moisture content of the soil and the latter may act as secondary responders (Lynd et al. 2002). Similarly, in decaying wood, fungi act together with bacteria, constituting the communities that collectively work on the substrate (Prewitt et al. 2014) whereas in sediment, we expect anaerobic cellulolytic bacteria, possibly concomitant with particular fungi, to be involved in the biopolymer degradation processes (Wei et al. 2009). Although cooperation between microbes seems to be the driving force behind lignocellulose degradation in natural habitats, in industry single strains are often used (Guerriero et al. 2015).

Using microbial consortia instead of single strains for the biodegradation of lignocellulose allows one to take advantage of the microbial interactions, by making optimal use of their intricate regulatory systems, which may bypass problems of feedback regulation and metabolite repression that are often posed by single strains (Wongwilaiwalin et al. 2010). Additionally, this strategy may confer an optimal combination of enzyme production and interaction of microbes. Enrichment culturing—also coined “habitat biasing” (Ekkers et al. 2012)—is a strategy in which a deliberate “bias” is introduced into an environmental sample in order to modulate the microbial community with respect to function (in situ or ex situ). The selective media that are used enhance the prevalence of desired functions in a microbial community and so the genes and/or operons of interest, as was shown for chitin (Cretoiu et al. 2012) as well as hemicellulose degradation processes (Jiménez et al. 2013).

Microbial consortia with effective lignocellulose degradation capacity can be selected from different source materials (Wongwilaiwalin et al. 2010; Wang et al. 2011; Jiménez et al. 2013; Moraïs et al. 2014), leading to effective and structurally-stable consortia that successfully degrade substrates even beyond the ones they were selected upon (Haruta et al. 2002). However, little is known about the differences that might arise when different microbial sources are used to breed such degrader consortia on the same substrate, i.e., whether microbial communities originating from different sources would converge to similar consortia when exposed to enrichment culturing. In that case, this convergence would provide evidence for the 100-year old Beijerinck adagium “Everything is everywhere and the environment selects”.

Here, we investigated whether different source communities would generate similar lignocellulolytic microbial consortia when similar selection criteria are applied. Whether this adagium would be turned into reality presumably depends on (1) the nature of the source inocula and (2) the strength of the selective force applied. Thus, the aims of this study were (i) to determine the relevance of the microbial inoculum as the driver of the lignocellulose-degradative consortia produced after ten enrichment steps and (considering the high degree of functional redundancy often observed in microbial communities) (ii) to assess whether different source inocula result in similar degradation capacities. To this end, three different source inocula, i.e. microbiomes from forest soil, canal sediment and decaying wood, were used for serial-batch dilution-to-stimulation on severed wheat straw as the carbon- and energy-yielding substrate, in order to measure their effects on the final enriched consortia.

## Materials and methods

### Substrate preparation

Wheat straw used as the substrate was obtained from a local farm (Groningen, the Netherlands). It was air-dried (50 °C) before cutting it into pieces of about 5 cm length. Then, the pieces were mixed with sterile distilled water and thoroughly ground, using a mill hammer, to pieces ≤1 mm in order to increase the surface to volume ratio. After this treatment, the wheat straw suspension was autoclaved at 121 °C for 27 min before use. Sterility of the substrate was verified following plating on LB agar plates.

### Selection of microbial consortia degrading wheat straw from three inoculum sources

The sources of the microbial communities used in this experiment were forest soil, decaying wood and canal sediment. Forest soil encompassed three randomly collected (53.41 N; 6.90 E) 10-g surface (0–10 cm) samples which were thoroughly mixed. The decaying wood was collected at the same site. A 20-cm decomposing tree branch (hardwood), with evident microbial growth on its surface, was used. In the laboratory, it was cut into small (<3 mm) pieces in sterile conditions. Sediment was collected from the bottom of an adjacent canal, using three random samples of about 10 g, which were thoroughly mixed. All samples were taken in February 2014 (winter season). Cell suspensions were prepared by adding 10 g of each of the microbial sources to 250 mL flasks containing 10 g of sterile gravel in 90 mL of mineral salt medium (MSM; 7 g/L Na_2_HPO_4_; 2 g/L K_2_HPO_4_; 1 g/L (NH_4_)_2_SO_4_; 0.1 g/L Ca(NO_3_)_2_; 0.2 g/L MgCl_2_, pH 7.2). All flasks were shaken for 30 min at 200 rpm (room temperature). To start the experiments, 250 μL of each cell suspension were added to triplicate 100-mL Erlenmeyer flasks containing 25 mL of MSM supplemented with 1 % (*w*/*v*) sterilized wheat straw, 25 μL of vitamin solution (0.1 g Ca-pantothenate, 0.1 g cyanocobalamin, 0.1 g nicotinic acid, 0.1 g pyridoxal, 0.1 g riboflavin, 0.1 g thiamin, 0.01 g biotin, 0.1 g folic acid; H_2_O 1 L) and 25 μl of trace metal solution (2.5 g/L EDTA; 1.5 g/L FeSO_4_; 0.025 g/L CoCl_2_; 0.025 g/L ZnSO_4_; 0.015 g/L MnCl2; 0.015 g/L NaMoO_4_; 0.01 g/L NiCl_2_; 0.02 g/L H_3_BO_3_; 0.005 g/L CuCl_2_). All chemicals and reagents used in this work were of analytic molecular biology grade (Sigma-Aldrich, Darmstadt, Germany). All flasks were incubated at 28 °C, with shaking at 200 rpm. The cultures were monitored by counting cells in a Bürker-Türk chamber at regular time intervals. At the start of the experiments, around 5 log cells/mL were used. Once the systems had reached around 9 log cells/mL (and straw had visually been degraded), 25 μL of culture was transferred to 25 mL of fresh medium (dilution factor 10^−3^). The procedure was repeated nine times, giving a total of ten sequential enrichment cultures. Following each transfer (T), part of the bred consortia was stored in 20 % glycerol at −80 °C. The consortia of the T1, T3, T6, and T10 flasks were used for all subsequent analyses, as detailed below. As controls, we used microbial sources in MSM without substrate (C1a, C1b, C1c) as well as MSM plus substrate without inoculum (C2a, C2b, C2c).

### DNA extraction and quantitative PCR (q-PCR)

Aliquots (2 mL) of each selected culture were used for community DNA extraction using the “Power Soil” DNA extraction kit (inoculum sources) (MoBio® Laboratories Inc., Carslab, USA) or the “UltraClean” DNA Isolation Kit (each enriched consortium) (MoBio® Laboratories Inc., Carslab, USA). The instructions of the manufacturer were followed, except that the resuspension of the DNA from the inoculum sources was in 60 μL resuspension fluid. The 16S rRNA gene region V5-V6 (bacteria), as well as the ITS1 region (fungi), were amplified using 1 ng of community DNA as the template and primers 16SFP/16SRP and 5.8S/ITS1(Pereira e Silva et al. 2012), respectively. Standard curves were constructed using serial dilutions of cloned 16S rRNA gene and ITS1 fragments from *Serratia plymuthica* (KF495530) and *Coniochaeta ligniaria* (KF285995), respectively. Gene target quantification was performed, in triplicate, in an ABI Prism 7300 Cycler (Applied Biosystems, Lohne, Germany).

### PCR-DGGE analysis

Total community DNA was used as the template for amplification of the partial 16S rRNA gene fragment using *Taq* DNA polymerase (Bioline, Lückenwalde, Germany) with primer F968 with a GC clamp attached to the 5′ end and universal bacterial primer R1401.1b. For ITS1 amplification, primers EF4/ITS4 were used; this PCR was followed by a second amplification with primers ITS1f-GCITS2. Primer sequences, the reactions mixtures, and cycling conditions have been described (Pereira e Silva et al., 2012). The DGGE was performed in 6 % (*w*/*v*) polyacrylamide gels with 45–65 % and 20–50 % denaturant gradients for bacterial and fungal communities, respectively (100 % denaturant is defined as 7.0 M urea with 40 % deionized formamide). Electrophoresis was carried out at 100 V and 75 mA, for 16 h at 60 °C. The gels were subsequently stained for 40 min in 0.5 % TAE buffer with SYBR gold (final concentration 0.5 μg/L) (Invitrogen, Breda, the Netherlands) (Fig. S1 in the Supplementary Material). Gel images were digitized using Imagemaster VDS (Amersham Biosciences, Buckinghamshire, UK). The DGGE patterns were then transformed to a band-matching table using GelCompar II software (Applied Maths, Sint Martens Latem, Belgium).

### Analysis of the three final consortia by sequencing of the 16S rRNA gene

Amplicons of 250 bp were generated on the basis of primers amplifying the V4-V5 of the 16S rRNA gene region. PCR amplifications were conducted in triplicate reactions for each of the 12 samples with the 515F/806R primer set (Table S1 in the Supplementary Material). PCR and sequencing were performed using a standard protocol (Caporaso et al. 2012). Illumina MiSeq sequencing was performing at Argonne National Laboratory (Illinois, USA). We processed the raw data using “quantitative insight into microbial ecology” (QIIME) software, version 1.91. The sequences were de-multiplexed and quality-filtered using split_libraries_fastq.py default parameters (Bokulich et al. 2013). The derived sequences were then clustered into operational taxonomic units (OTU) using open-reference OTU picking against the Greengenes reference OTU database with a 97 % similarity threshold (Rideout et al. 2014). Then, we performed quality filtering to discard OTUs present at very low abundance (<0.005 %) (Bokulich et al. 2013). An even sampling depth of 10,000 sequences per sample was used for assessing α- and β-diversity measures by using core_diversity_analyses.py. Metrics for α-diversity were OTU richness (equivalent to species richness), Chao1 index (estimated species richness) and Faith’s phylogenetic diversity (PD) index (phylogenetic relationship between OTUs). β-diversity analyses among the final consortia were performed using unweighted UniFrac distance matrix (Lozupone et al. 2011). Statistical analyses, i.e. matrix similarity and principal coordinate analyses (PCoA) and UniFrac were performed with the PREMIER 6 and PERMANOVA A+ software packages (Primer-E Ltd., Lutton, United Kingdom).

### Substrate degradation analysis in the consortia

After each growth step, the remaining particulate wheat straw was recovered from the microcosm flasks, after which this material was washed to remove microbial cells. The degradation rates of the components of the substrate, before and after incubation, were determined by Fourier-transformed infrared (FTIR) spectra (Adapa et al. 2011; Xu et al. 2013) and partial least squares (PLS) regression. Spectra were obtained with a resolution of 4 cm^−1^ from Perkin Elmer Spectrometer FTIR (model UATR, version Two). Thirty-two scans were run per sample between 800 and 1800 cm^1^ (Krasznai et al. 2012). Each sample (calibration and consortium samples) was analyzed in triplicate. Before PLS regression, all spectra were subjected to baseline correction and then corrected for physical effects by 2nd derivative Savitzky-Golay (FitzPatrick et al. 2012). A model was created on the basis of a calibration with standard mixtures, consisting of hemicellulose (proxy Beechwood xylan, ≥90 %, Sigma Aldrich, Steinheim, Germany), cellulose (powder, D-516, Macherey-Nagel, Düren, Germany), and lignin (alkaline, Sigma Aldrich, Steinheim, Germany) in the proportion described in Table S2 in the Supplementary Material (Adapa et al. 2011). The model displayed *R*^2^ values of 0.95, 0.97 and 0.99 for hemicellulose, cellulose and lignin, respectively. Correction and analysis of the spectra were conducted using Unscrambler X by CAMO software (FitzPatrick et al. 2012; Krasznai et al. 2012). All FTIR measurements were carried out on oven-dried material (50 °C, 24 h). The degradation of hemicellulose components was calculated by subtracting the percentage of the residual substrate from the total percentage of each hemicellulose component before degradation. Degradation rate was calculated using the followed equation: $\frac{Ci-Cf}{Ci}x100$, where *C*_*i*_ is the total amount of compound before degradation and *C*_*f*_ is the residual component after degradation (Wang et al. 2011). One-way analysis of variance (ANOVA) followed by Tukey’s HSD pairwise group comparisons was performed in IBM SPSS Statistics version 23 (SPSS, Illinois, USA).

### Quantification of enzymatic activities related to (hemi)cellulose degradation in the consortia

Using the extracellular fractions (containing the “secretome”) of the three final consortia (T10), the specific activities of β-xylosidases, β-galactosidases, β-mannosidases, cellobiohydrolases and β-glucosidases were measured. To do so, microbial cells and wheat substrate were harvested by centrifugation (5 min, 13,500 rpm; Thermo Fisher Scientific Centrifuge; Thermo Fisher, Waltham, USA), after which the supernatants were used directly in the tests. MUF-β-D-xylopyranoside, MUF-β-D-mannopyranoside, MUF-β-D-galactopyranoside, MUF-β-D-cellobioside, and MUF-β-D-glucopyranoside were used as the fluorogenic substrates (Sigma-Aldrich, Darmstadt, Germany). The reaction mixes consisted of 10 mM MUF-substrate in dimethyl sulfoxide, 15 μL Mcllvaine buffer (pH 6.8) and 25 μL of supernatant. The reaction was incubated at 25 °C for 45 min in the dark, after which it was stopped by adding 150 μL of glycine–NAOH buffer (0.2 M, pH 10.4). Fluorescence was measured at an excitation wave length of 365 nm and with emission at 445 nm. Enzymatic activities were determined from the fluorescence units using a standard calibration curve. We then determined total protein using the DC Protein Assay (BioRad, Hercules, USA) according to the manufacturer’s instructions. The specific enzymatic activity was reported as the rate of MUF production (μM MUF per min per mg at 25 °C, pH 6.8).

### Identification and phylogenetic analysis of bacterial and fungal strains

From the three final degrader consortia, we isolated bacterial and fungal strains, using R2A (BD Difco®, Detroit, USA) and Potato Dextrose Agar (PDA) (Duchefa Biochemie BV, Haarlem, The Netherlands), respectively. The isolation part can be found in Electronic supplementary material (ESM 1). For the identification of bacterial strains, primer 1406 was used (sequencing the 16S rRNA gene), whereas for fungal identification primer ITS4 was used (sequencing a partial region of the 18S rRNA gene) (Jiménez et al. 2013). After, the amplicons were sequenced by Sanger technology (LGC Genomics, Lückenwalde, Germany). All sequence chromatograms were analyzed for quality (Brossi et al. 2015). Taxonomic assignments of the sequences were done by using BLAST-N against the NCBI database (http://blast.stva.ncbi.nlm.nih.gov/Blast.cgi). We used the best BLAST hit affiliation for taxonomic assignment with a cut-off of 97 % (identity) and 95 % (coverage). Sequences are publicly available in the GenBank database under accession numbers KT265747 to KT265810 (Tables S3 and S4 in the Supplementary Material).

### Matching bacterial strains with abundant OTUs

The recovered bacterial strains were linked to the OTUs based on sequence similarity and clustering. The almost-full-length 16S rRNA gene sequences from the strains were compared—in the specific V4 region—to sequences of the abundant OTUs using ClustalW. Phylogenetic analyses (*p*-distance) were conducted with MEGA v6 using Neighbor Joining. Evolutionary distances were computed using the Kimura-2 parameter method. The branch node strengths were tested with bootstrap analyses (1000 replications) (Fig. S2 in the Supplementary Material). Additionally, we also matched the presence of the bacterial strains in the final consortia by comparing their patterns to those observed with consortium PCR-DGGE (Fig. S3–S5 in the Supplementary Material).

## Results

### Effective wheat straw degrading microbial consortia produced from three different inoculum sources

**Fig. 1:**
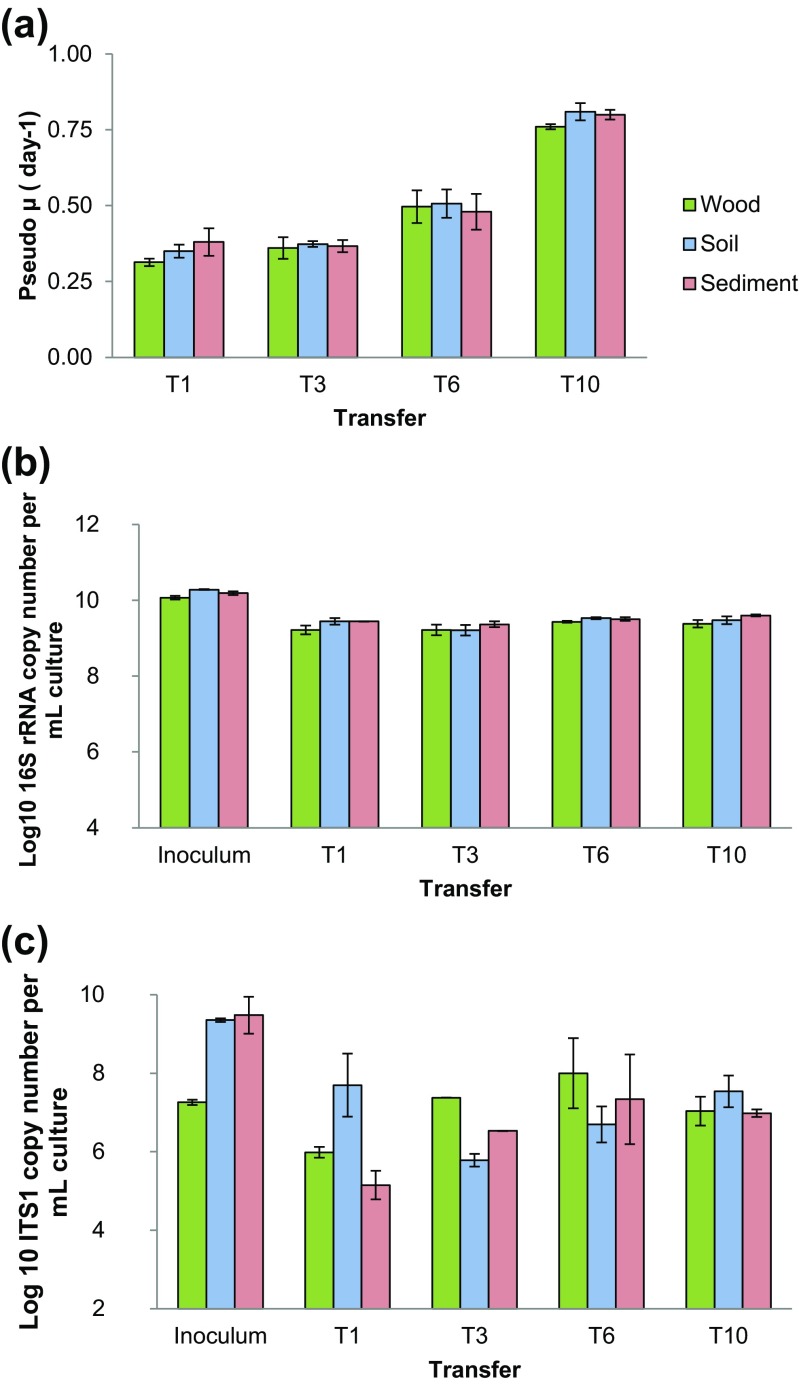
Sequential-batch selection of lignocellulose-degradative microbial consortia from three inoculum sources: decaying wood (*green*), forest soil (*blue*) and canal sediment (*red*). Bacterial and fungal growth rates increased along the transfers, which consisted of additions of inoculum (rate 1:1000) to each fresh medium. Data were collected after four transfers. The *x*-axis shows the transfer number, the *y*-axis represents **a** pseudo μ, rate of increase of bacterial cell, **b** 16S rRNA gene copy numbers, **c** ITS1 copy numbers (both: log copies per mL) determined by qPCR. *Bars* refer to standard errors of the mean (*n* = 3). The three different microbial sources (Inoculum) were used as inocula for starting the enrichment process

For all inocula used, growth took place in each flask (28 °C, with shaking), yielding well-developed microbial consortia at the end of each growth step. The three consortia were found to progressively raise their overall fitness (measured as average growth rate) along the transfers, as a progressive reduction of the incubation time necessary to reach maximal cell densities was recorded. Specifically, we found a significant increase in the growth rates of the final consortia (T10) as compared to those of the previous transfers (Fig. 1a). In contrast, the without-substrate negative control C1 revealed cell numbers that progressively decreased from 6 to 2 log cells/mL at T3, thereafter remaining below the detection limit (data not shown). The negative controls without added inoculum did not reveal the presence of any cells along the transfers.

The microscopic cell counts were corroborated by the 16S rRNA gene and ITS1 copy numbers (proxies for bacterial and fungal communities, respectively), measured in the T1, T3, T6 and T10 consortia (Fig. 1b, c). These results revealed that, at the end of each transfer, the three consortia reached maximal (bacterial) levels on the order of 9 log 16S rRNA gene copy numbers per mL, which was consistent with the aforementioned cell counts (Fig. 1b). Additionally, progressively lower numbers were detected in the (without-substrate) control flasks C1 from T1 to T3, revealing that any major growth was absent from the systems without added wheat straw (data not shown). Concerning fungal abundances, the numbers of ITS1 gene copies at the end of the first transfer, in all consortia, showed a marked reduction compared to those of the inoculum sources. However, along the transfers, these numbers increased slightly, reaching the maximal number in the last transfer. In detail, these numbers were 6.0 log ±0.1 (T1), 6.8 log ±0.4 (T10), and 5.1 log ±0.2 (T1) and 7.0 log ±0.1 (T10), for wood- and sediment-derived consortia, respectively. In the case of the soil-derived microbial consortium, a population size decrease occurred at T3 (5.8 log ±0.2), after which 7.4 log ±0.4 was reached at T10 (Fig. 1c).

### Analysis of the wheat straw degrading microbial consortia

Overall, the data clearly yielded evidence for the contention that inoculum source primarily determines the structure of the final effective consortia. The three microbial consortia were first analyzed by bacterial- and fungal-specific PCR-DGGE analyses, on the basis of the directly-extracted consortial DNA. The consortia revealed considerable changes in structure over time, as evidenced by reductions in the band numbers in the DGGE patterns for both the bacterial and fungal communities. The bacterial banding patterns were consistent between the triplicates per treatment, indicating reproducibility within the treatment in terms of consortium structure build-up. The fungal patterns, however, showed higher dissimilarity between treatments and transfers (Fig. S1 in the Supplementary Material).

**Fig. 2:**
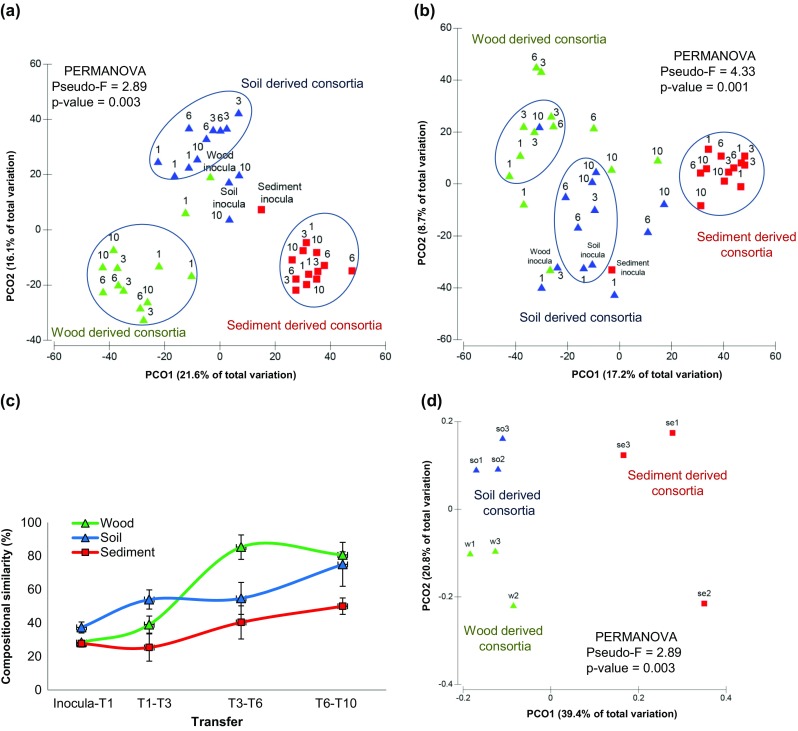
Analyses of steps of the enrichment process and dynamics of the selected consortia*.* Principal coordinates analysis (PCoA) revealing well-defined clusters along microbial inoculum, especially for the bacterial communities (the fungal communities were less clearly differentiated). **a** bacterial and **b** fungal communities obtained from the PCR-DGGE abundance data. The final communities (T10) for both bacteria and fungi are well grouped and differentiated from their respective source communities, indicating unique compositions depending on inoculum source. **c** moving window analysis (MWA). Evaluation of the community divergence between two sequential transfers in the enrichment process, as measured by percentage of similarity. MWA shows how the communities evolve through the enrichment process. Consortia: wood-derived (*blue line*), soil-derived (*green line*) and sediment-derived (*red line*). **d** Principal coordinates analysis (PCoA) of unweighted UniFrac distances for 16S rRNA gene sequencing data of final (T10) enrichment cultures (61-day incubation time) from decaying wood (*green triangle*), forest soil (*blue triangle*), and canal sediment (*red square*) inocula. Ordination of bacterial communities shows strong separation with respect to community composition depending on the microbial source used as inoculum

Principal coordinate analysis of the bacterial (Fig. 2a) and fungal community patterns (Fig. 2b) based on DGGE profiles indicated (i) a clear clustering along inoculum source, (ii) separation of all patterns from the initial (inoculum) ones, and (iii) a progressive evolution with time, with persisting clustering along the microbial source. Moreover, PERMANOVA indicated the existence of significant differences between the consortia between the treatments for both bacteria (*P* < 0.005) (Fig. 2a) and fungi (*P* < 0.005) (Fig. 2b).

Moving window analysis showed that the similarity between the bacterial community structures increased along the transfers. This was true for all consortia, i.e. those from wood, soil and sediment (Fig. 2c). Altogether, the data indicated a reduction in the diversity of the bacterial communities throughout the transfers. Specifically, according to the similarity percentage between the communities between T6 and T10 (higher similarity indicates greater stabilization), the consortia reached stabilization in the following order: wood-derived (81 % ± 2), soil-derived (75 % ± 13) and sediment-derived (50 % ± 5) ones. The consortia derived from wood revealed only few changes in their structures at an intermediate time point (T6) compared with the other two consortia, whereas the soil-derived consortia reached stabilization between T6 and T10. In contrast, the sediment-derived consortia did not reach a plateau. Thus, stabilization was clearly achieved for the wood-derived but less so for the soil- and sediment-derived consortia.

### Detailed analysis of the bacterial consortia

**Fig. 3:**
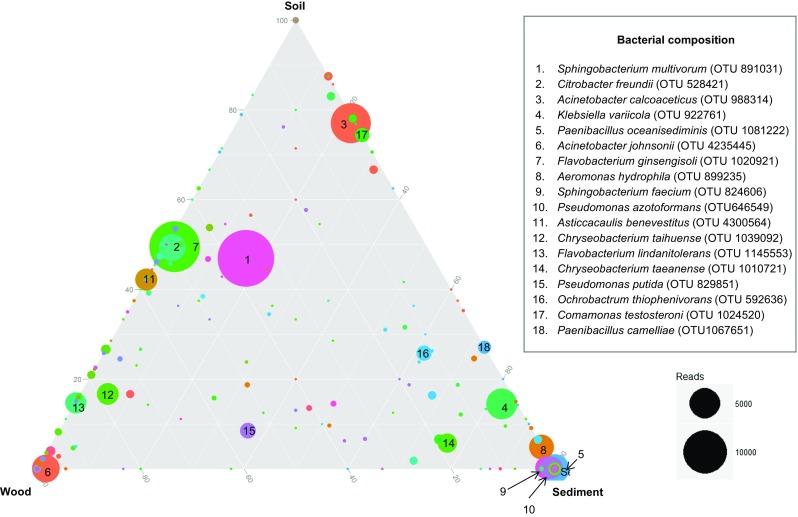
Differences in composition of the three final consortia bred from three different inocula. Ternary plot of OTU relative abundances. A common bacterial core is formed by members of the genera *Sphingobacterium*, *Acinetobacter* and *Chryseobacterium.* Resolution level of 800 reads

Direct amplicon sequencing revealed that the bacterial richness values of the final consortia (wood, soil and sediment derived) were 241.7 ± 34.2, 227.7 ± 11.6, and 137.7 ± 19.7 OTUs, respectively, indicating that the final sediment-derived consortia were less rich than the other ones (ANOVA, *P* < 0.05). Regarding the bacterial community structures (β-diversities), PCoA of the unweighted UniFrac community distances showed that the final consortia (T10) were markedly different between the treatments (Fig. 2d). Moreover, PERMANOVA showed that the structure of the bacterial communities in these consortia was significantly affected by the inoculum source (*P* < 0.005). Specifically, more similar structures were found between the soil- and wood-derived consortia (0.42 ± 0.03), indicating that these two environments share comparable microbiomes. This was corroborated by the fact that the wood- (0.558 ± 0.042) and soil-derived consortia (0.558 ± 0.059) shared equal similarity to the sediment-derived ones. Delving into taxonomic affiliations (using OTUs with abundance >2 %) revealed that members of three bacterial genera, i.e. *Sphingobacterium*, *Acinetobacter* and *Chryseobacterium*, constituted a “core”-type community that was present across all replicates of the three final consortia*.* The relative abundance (%) of *Sphingobacterium* in the three consortia was 18.9 ± 1.8, 24.4 ± 4 and 16.6 ± 0.3, that of *Acinetobacter* was 14.7 ± 9.2, 22.2 ± 5.9 and 7.8 ± 7.8, and that of *Chryseobacterium* was 6.9 ± 7.9, 1.7 ± 0.7 and 7.9 ± 6.2, for the wood-, soil- and sediment-derived consortia, respectively (Fig. 3).

Each of the final consortia further revealed “unique” (only occurring in that type of consortium) microbiome members, i.e. the soil-derived consortia exclusively contained OTU 1024520, which was associated with *Comamonas testosteroni* (2.5 ± 0.2), the sediment-derived consortia had members of the genera *Paenibacillus* (OTU 1081222, *P. oceanisediminis*, 13.5 ± 9.6; OTU 1067651, *P. camelliae*, 2 ± 2.9), *Aeromonas* (OTU 839235, *A. hydrophila*; 9.1 ± 9.2), and *Ochrobactrum* (OTU 592636, *O. thiophenivorans*, 2.2 ± 3.2) (Table 1, Fig. 3). In the wood-derived consortia, we found high abundances of the genera *Pedobacter* (OTU 106847, *P. agri*, 0.4 ± 0.4), and *Taibaiella* (OTU 771274, *T. koreensis*, 1.2 ± 1.2); however, the unique OTUs were found in low relative abundances.

**Table 1:** Most abundant OTUs in the final microbial consortia derived from decaying wood, forest soil, and canal sediment

Consortia derived from	OTU (Identificator)	Taxonomic affiliation	Relative abundance (%)
Decaying wood	1145553	*Flavobacterium lindanitolerans*	6.0 ± 7.7
	4300564	*Asticcacaulis benevestitus*	4.4 ± 3.1
	1039092	*Chryseobacterium taihuense*	5.8 ± 6.8
	528421	*Citrobacter freundii*	19.3 ± 5.1
	891031	*Sphingobacterium multivorum*	18 ± 11
	1020921	*Flavobacterium ginsengisoli*	5.6 ± 2.2
	829851	*Pseudomonas putida*	2.2 ± 1.1
	4235445	*Acinetobacter johnsonii*	11.8 ± 7.6
Forest soil	1024520	*Comamonas testosteroni*	2.4 ± 0.2
	4300564	*A. benevestitus*	3.2 ± 4.5
	528421	*C. freundii*	19.7 ± 3.9
	891031	*S. multivorum*	23.4 ± 3.7
	1020921	*F. ginsengisoli*	5.7 ± 1.2
	988314	*Acinetobacter calcoaceticus*	19.3 ± 5.3
	922761	*Klebsiella variicola*	2.1 ± 1.5
Canal sediment	646549	*Pseudomonas azotoformans*	7.6 ± 10.6
	1081222	*Paenibacillus oceanisediminis*	13.5 ± 9.6
	839235	*Aeromonas hydrophila*	9.1 ± 9.2
	592636	*Ochrobactrum thiophenivorans*	2.2 ± 3.2
	746501	*Chryseobacterium taichungense*	2.5 ± 2.2
	1067651	*Paenibacillus camelliae*	2 ± 2
	891031	*S. multivorum*	8.4 ± 11.8
	824606	*Sphingobacterium faecium*	8.2 ± 11.5
	1010721	*Chryseobacterium taeanense*	4.5 ± 4
	988314	*A. calcoaceticus*	5.5 ± 7.1
	922761	*K. variicola*	12.3 ± 6.8

Similarity between the OTU 16S rRNA gene sequence and the taxonomic affiliation as in NCBI

### Substrate degradation patterns and enzymatic profiles of the final microbial consortia

**Fig. 4:**
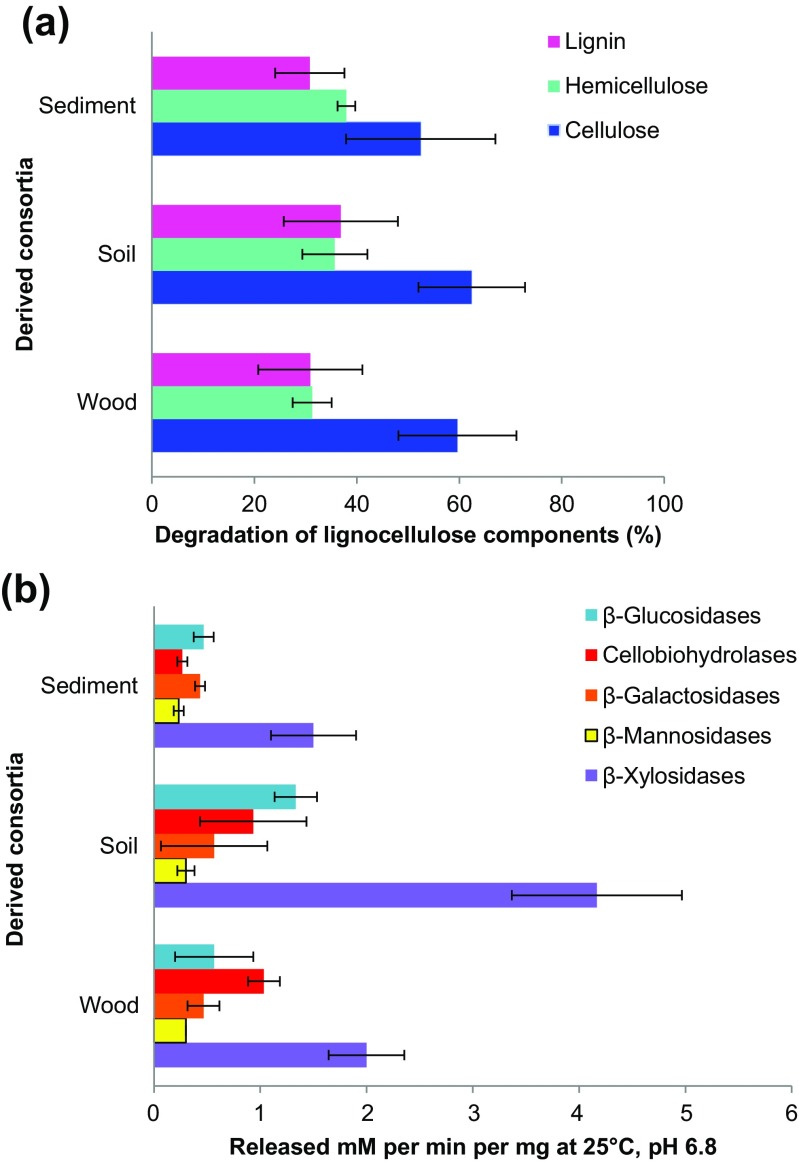
Degradation capacity profiles of **a** Percentage of reduction of hemicellulose, cellulose and lignin, after incubation with the different microbial consortia. **b** Quantification of enzymatic activities by methyl umbelliferyl (MUF)-substrate, measured in the recovered culture supernatants

The final consortia consumed the hemicellulose, cellulose, and lignin components of the substrate to grossly similar extents, as only small and insignificant differences were found between them (ANOVA, *P* > 0.05) (Fig. 4a). The variation levels prevented the drawing of strong conclusions with respect to the degradation efficacies. All final consortia were found to preferably consume the hemicellulose part of the substrate, which was more than 50 % degraded. With respect to cellulose and lignin, the degradation rates were lower throughout. Interestingly, there was a trend in that the sediment-derived consortia had a subtle but non-significant lower hemicellulose and a higher cellulose degradation rate compared with the soil- and wood-derived consortia. The enzymatic profiling revealed that each of the three final consortia (T10) had a unique combination of specific enzymatic activities (Fig. 4b). Remarkably, the sediment-derived consortia showed a more even distribution of the activities, whereas the soil- and wood-derived consortia were dominated by β-glucosidases and cellobiohydrolases, respectively. Notably, β-xylosidase was the enzyme with the highest activity in all treatments. The high β-xylosidase activities corroborated the observation that the main degradation activity was on the hemicellulose part of the substrate.

### Biodegradative bacterial and fungal strains from the wheat straw bred microbial consortia

In total, 90 bacterial strains were recovered from the three final consortia, 52 of which were identified on the basis of 16S rRNA gene sequencing. Using co-migration analysis, several DGGE bands were presumptively identified as being derived from several strains (Fig. S3–S5 in the Supplementary Material), indicating that these strains contributed to the dominant bacterial species present in the consortia. The strains were subsequently screened with respect to various enzymatic activities providing them with the ability to degrade lignocellulose. This was indeed a widespread characteristic across the strains, as 70 % showed enzymatic activity for at least two out of six enzymes tested (Table S3 and Fig. S6 in the Supplementary Material).

By aligning the 16S rRNA gene sequence from the bacterial isolates with the OTUs obtained by sequencing the whole consortia (Table 2), we could verify that several strains that were highly abundant in the three consortia (according to the higher number of sequences for each specific OTU) presented key enzymatic activities (Table 2). These strains were *Sphingobacterium multivorum* soB22, wB15 and seB10, *Citrobacter freundii* soB4, *Lelliotia amnigena* soB12 and seB4, *Flavobacterium ginsengisoli* wB6 and soB9, *Chryseobacterium taihuense* wB4 and soB3, *Asticcacaulis benevestitus* wB3 and *Ochrobactrum thiophenivorans* soB16. Moreover, from the three final consortia, we obtained several biodegradative strains showing α-D-glucosidase, β-D-glucosidase, β-D-galactosidase, and β-D-xylosidase activities, although none of these dominated in the consortia according to the OTU sequencing results (Table 2). These strains were affiliated to *Stenotrophomonas terrae* (wB16), *S. rhizophila* (seB11), and *Microbacterium foliorum* (wB9).

**Table 2:** Molecularly-identified organisms in lignocellulolytic consortia bred from decaying wood, forest soil, and canal sediment

		Wood	Soil	Sediment									
Class	OTU	RA (%)	RA (%)	RA (%)	Similarity (%)	Isolated bacteria strain^a^ (strain code)	A	B	C	D	E	F	Accession number
*Sphingobacteria*						*Sphingobacteriales*							
	OTU 891031 *S. multivorum*	18 ± 11	23.4 ± 3.6	8.4 ± 11.8	100	*S. multivorum* (soB22)	+	+	+	+	+	+	KT265757
					100	*S. multivorum* (wB15)		+	+	+			KT265760
	OTU 824606 *S. faecium*	<2.0	0	8.2 ± 11.5	100	*S. faecium* (seB10)	+	+	+	+			KT265798
*Gammaproteobacteria*						*Enterobacteriales*							
	OTU 528421 *C. freundii*	19.3 ± 5.2	19.7 ± 3.9	0	100	*C. freundii* (soB4)				+			KT265771
	OTU 922761 *K. variicola*	<2.0	2.1 ± 1.5	12.3 ± 6.8	100	^b^ *Lelliottia amnigena* (soB12)	+	+		+			KT265765
					100	*L. amnigena* (seB4)	+	+		+			KT265772
					100	^b^ *Raoultella terrigena* (wB13)	+	+		+			KT265749
					100	*R. terrigena* (soB20)	+	+		+			KT265778
					100	*R. terrigena* (seB9)	+	+		+			KT265755
	OTU 569939 *S. terrae*	<2.0	<2.0	0	100	*S. terrae* (wB16)		+		+			KT265788
	OTU 544847 *S. rhizophila*	<2.0	<2.0	<2.0	100	*S. rhizophila* (seB11)	+						KT265763
*Flavobacteria*						*Flavobacteriales*							
	OTU 1020921 *F. ginsengisoli*	5.6 ± 2.2	5.7 ± 1.2	<2.0	100	*F. ginsengisoli* (wB6)	+			+			KT265776
					100	^c^F. *ginsengisoli* (soB8)	+	+		+			KT265787
	OTU 1039092 *C. taihuense*	5.8 ± 6.8	<2.0	<2.0	100	^d^ *C.taihuense* (wB4)	+	+	+	+	+		KT265756
					100	^d^ *C. taihuense* (soB3)	+						KT265758
*Alphaproteobacteria*						*Caulobacterales*							
	OTU 4300564 *A. benevestitus*	4.4 ± 3.1	3.2 ± 4.6	<2.0	100	*A. benevestitus* (wB3)		+		+	+		KT265751
						*Rhizobiales*							
	OTU 592636 *O. thiophenivorans*	<2.0	<2.0	2.3 ± 3.2	100	*O. thiophenivorans* (soB16)	+	+	+		+		KT265790

Affiliation is taxonomic showed Class, order, and species level of the isolated bacterial strains. Similarity (%) related between the OTU sequence and the 16S rRNA from isolated recovered strains

*RA* relative abundance; Enzymatic activities: *A* α-D-glucosidase, *B* α-D-glucosidase, *C* α-D-mannosidase, *D* β-Dgalactosidase, *E* β-D-xylosidase, *F* α-L-fucosidase

^a^Closest relative species according to 16S ribosomal RNA gene sequence

^b^Due to the high similarity in this family the multiple alignment of the analyses sequence region is the same

^c^BLAST analysis of the strain soB8 identified as *F. banpakuense*, however, multiple sequencing alignment indicated a perfect match with the OTU1020921 affiliated to *F. ginsengisoli*

^d^BLAST analysis of the strains wB4 and soB3 identified as *Chrysobacterium hagamense*, however, multiple sequencing alignment indicated a perfect match with the OTU1039092 affiliated with *C. taihuense* (Fig. S2)

Interestingly, some closely-related strains (in some cases identified as the same species) isolated from the different consortia expressed different enzymatic activities. For instance, two *S. multivorum* strains, i.e. wB15 and soB22, *F. ginsengisoli* strains wB6 and soB8 and *C. taihuense* strains wB4 and soB3, recovered from the wood- and soil-derived consortia, respectively, revealed completely different enzymatic profiles (Table 2). These results indicate that each final consortium constitutes a unique community in which each member, even the same species, participates potentially with a strain-unique set of enzymes for the degradation of the lignocellulose.

Regarding the 40 fungal strains, partial ITS1 sequence analyses revealed that they belong to 11 different species. Testing the fungal strains for (hemi) cellulolytic activity in media with CMC, xylan and cellulose as the single carbon sources revealed extracellular enzyme activities in most of them. Fungal strains from soil- and sediment- derived consortia presented the highest enzymatic activities, whereas only four strains isolated from wood had considerable activity in all the substrates. Moreover, two strains, wF4 and wF5 (associated with the taxa *Exophiala* and *Herpotrichiellaceae*, respectively), did not show any activity; the strains did not grow on glucose as a single carbon source (used as a positive control) (Table S4 and Fig. S7 in the Supplementary Material).

## Discussion

Microbial consortia have been proposed as a reliable and efficient alternative to single strains for lignocellulose degradation purposes (Jiménez et al. 2013; Brossi et al. 2015). When creating such consortia—usually achieved via dilution-to-stimulation approach—the source of the inoculum might determine the effectiveness of the final community. In this study, we addressed the question whether breeding different inocula on the same carbonaceous substrate, i.e. suspended severed wheat straw, would yield taxonomically and functionally similar microbial consortia. We used inocula from forest soil, decaying wood and canal sediment, and analyzed the nascent microbial consortia over time by cultivation-based as well as direct molecular approaches. Clearly, regarding the functioning of the consortia (i.e. degradation of, and growth on, wheat straw as carbon and energy source), high similarity was found. The results thus touch upon two classical paradigms in microbial ecology, i.e. (1) Beyerinck’s postulate “everything is everywhere” and (2) the functional redundancy across and within microbial communities. Overall, our data showed the three microbial consortia to be taxonomically quite different, with a small core community being detectable across them (at genus level). Thus, we cogitated that, within the confines of the experiment, microbial source rather than “environment” was the key driver of the composition of the final consortia, next to their intrinsic degradation and metabolic capacities. Overall, in terms of lignocellulose degradation, such consortia revealed similar rates. Thus, different bacterial and fungal key players had likely been selected from the diverse pools of microorganisms, performing similar functions under the condition applied. In their local habitats, such communities are influenced by conditions like water availability, oxygen availability, redox potential, temperature and available nutrients (Wei et al. 2009; Montella et al. 2015). Thus, the dissimilarities between conditions reigning in the forest soil, decaying wood and canal sediment habitats, resulting in presumably widely divergent microbiomes, may be at the basis of the differences seen, even after ten 1:1000 transfers in wheat straw batch cultures. In other words, such historical contingencies were not overwhelmed, in taxonomical terms, by the selection applied.

Regarding their degradation capacity**,** each microbial consortium showed an overall similar degradation pattern (Fig. 4a) but different enzymatic activity profiles (Fig. 4b). Thus, despite the overall functional redundancy regarding lignocellulose degradation, where the overall process rate was similar, the snapshot-like activity profiles differ. The degradation patterns in the final consortia were likely linked to the particular microbial compositions, as each organism likely contributed with different enzymes attacking the substrate (Table 2, Table S3 in the Supplementary Material). A remarkable finding was the fact that some bacterial strains, identified as the same or very closely related species, had completely different enzymatic palettes and that such differences were linked to the microbial source (Table 2).

Recently, Wongwilaiwalin et al. (2013) also compared the composition of bacterial consortia selected on the same substrate from different microbial inocula. The three consortia bred by them had similar composition at the phylum but were different at the genus level. Our findings stand in contrast to these, which may be attributed to differences in the enrichment conditions: whereas we used mesophilic temperature and mainly oxic conditions, they used high temperature, partial delignified substrate and anoxic conditions. Our findings, next to those of Wongwilaiwalin et al. (2013), showed the relevance of the inoculum, substrate selection and the culture condition for the final composition of the resulting consortia.

In spite of the fact that the three microbial consortia acted in a roughly similar overall manner on wheat straw (Fig. 4a), each revealed different sets of organisms and potentially different secreted enzymes working on the substrate. Wei et al. (2009) proposed different stages of increasing complexity in the microbial lignocellulose degradation process, where the degraders use a plethora of enzymes, in different combinations (Himmel et al. 2010; Moraïs et al. 2014). From the four major enzymatic realms that were invoked, i.e. free, cell-bound, multifunctional and cellulosome-bound enzymes (Bayer et al. 2013), the first two classes are thought to play major roles in our systems. Although we expect such enzymes to be working synergistically, this remains to be tested.

We here propose that *S. multivorum* (OTU 891031) has an important contribution to the degradation process in both the wood- and soil-derived consortia, as it was present in high abundance and—albeit in isolation—showed high degradation potential (Table 2)*.* Interestingly, in the sediment-derived consortia, next to *S. multivorum* (OTU 891031), two other strains likely were prominent contributors to the biodegradation process, i.e. *S. faecium* (OTU 824606) and *P. oceanisediminis* (OTU 1081222). The latter was the most abundant species; it has recently been reported as an important lignocellulose degrader (Liang et al. 2014)*.*

Regarding the fungi, several previous studies have described the lignocellulose-biodegrading capacities of both *Ascomycota* (Guerriero et al. 2015) and *Basidiomycota* (Rytioja et al. 2014). For instance, *Trichoderma reesii* can produce a highly efficient set of enzymes for the degradation of cellulose (van den Brink and de Vries, 2011). In contrast, *Aspergillus* species produce mainly enzymes for pectin degradation (van den Brink and de Vries 2011; Guerriero et al. 2015). Although we predict the involvement of fungi in wheat straw degradation, it was difficult to define the relative contribution of these organisms within our final consortia. Also, the selection of fungi was found to be highly dependent on the inoculum source and on their capacities to thrive in liquid (shaking) cultures (Wongwilaiwalin et al. 2010; Jiménez et al. 2013; Simmons et al. 2014). However, we surmised that, in our consortia, fungal-secreted degrading enzymes may have worked in conjunction with the bacterially-released ones.

The results of this study add another piece of evidence to the within-species diversity issue. The Beyerinck “everything is everywhere” paradigm may be expanded with the addition: “but not everything that is dissimilar performs in dissimilar ways.” Organisms that were shared across the microbial sources thus may have been involved in the degradation processes, but the overall process may have been supported by additional other organisms. Moreover, and rather surprisingly, taxonomically similar organisms may have been involved in different steps of the process, even within the species. Accordingly, the efficiency of the degradation process is related to the physiological adaptation and ecological niches of some of the consortial members in their original environment. Additionally, our results indicated that functional redundancy acts upon different levels, as all final consortia presented the same function (ability to degrade the substrate) but the relative contribution of each enzyme to the overall degradation process was probably different.

This study revealed that inoculum source was the strongest driver of the composition of the wheat straw degrading consortia that were produced over ten sequential-batch enrichments. Conspicuous differences emerged between the three consortia, next to similarities, leading to the concept of a core bacterial community that was shared. In functional terms, mixtures of enzymes, with, collectively, grossly similar joint capacities, were probably produced. In future work, the consortial secretomes, next to those from individual strains, may be used as sources of enzymes in the quest to maximize the production of sugars from the complex wheat straw.

## Electronic supplementary material

ESM 1 (PDF 1082 kb)
