# Supplementary material for: Different inocula produce distinctive microbial consortia with similar lignocellulose degradation capacity
Source: Appl Microbiol Biotechnol. 2016 May 12;100:7713–25. doi: 10.1007/s00253-016-7516-6 (PMC4980425; doi:10.1007/s00253-016-7516-6)
Supplement: Supplementary file 1 — (PDF 1082 kb) [file 253_2016_7516_MOESM1_ESM.pdf]

Applied Microbiology and Biotechnology

"Different inocula produce distinctive microbial consortia with similar lignocellulose degradation capacity"

Larisa Cortes-Tolalpa[a*], Diego Javier Jiménez[a], Maria Julia de Lima Brossi[a], Joana Falcao Salles[a], Jan Dirk van Elsas[a].

Genomics Research in Ecology and Evolution in Nature, Groningen Institute for Evolutionary Life Sciences, University of Groningen, Groningen, The Netherlands[a]

*Corresponding author: Larisa Cortes-Tolalpa, l.cortes.tolalpa@gmail.com, Nijenborgh 7, 9747 AG, Groningen, The Netherlands, +31 50 363 2236.

ELECTRONIC SUPPLEMENTARY MATERIAL AND FIGURES

Table S1 Primers for paired-end 16s community sequencing on the Illumina MiSeq platform. Bacteria primer 515F/806R. Forward primer 515F (GTGCCAGCMGCCGCGGTAA). Each reverse primer806R sequence contains different barcode

Table S2 Cellulose, hemicellulose (xylan) and lignin mixtures used to obtain the prediction. model

Table S3 Identification and enzymatic activities of bacterial strains isolated from final consortia: wood, soil and sediment derived consortia, obtained using three different inocula. Enzymatic activities: A = glucosidase; B= glucosidase (β); C = mannosidase; D = galactosidase (β); E = xylosidase (β); F = fucosidase. Bacteria strains isolated from wood, soil and sediment derived consortia, wB, soB, sedB, respectively. Closest relative specie. According to 16S ribosomal RNA gene.

Table S4 Identification and degradation activities of fungal strains isolated from final consortia: wood, soil and sediment derived , obtained using three different inocula.

ESM 1 Supplementary methods: Isolation of bacterial and fungal strains and hemicellulosic and cellulosic screening of isolated strains

Fig. S1 Analyses of steps of the enrichment process. PCR-DGGE analyses of **a)** bacterial and **b)** fungal communities at different transfer steps (T1, T3, T6 and T10). The DGGE patterns showed a reduction in the number of bands over experimental time for each of the three inocula. M: marker

Fig. S2 Neighbor Joining tree based on the comparison of 16S rRNA gene sequences from bacterial recovered strains and the most abundant OTUs in the final consortia from wood, soil and sediment inocula. Bootstrap values are expressed as percentages of 1000 replications. The scale bar estimates the number of substitutions per site

Fig. S3 Co-migration DGGE of enriched wood derived consortia community (T10) and recovered bacteria strains

Fig. S4 Co-migration DGGE analysis of enriched soil derived consortia community (T10) and recovered bacteria strains

Fig. S5 Co-migration DGGE analysis of enriched sediment derived consortia community (T10) and recovered bacteria strains

Fig. S6 Enzymatic activity detection by chromogenic substrate, in active bacterial strains isolated from final wood, soil and sediment derived consortia

Fig. S7 Enzymatic activity detection in CMC, xylan, and cellulose of fungal isolated from final wood, soil and sediment consortia

Table S1

SampleID	Reverse Primer	BarcodeSequence	LinkerPrimerSequence	Source	Transfer	Order	Time_day	Season	Description
W	806rcbc364	CACACAAAGTCA	GTGTGYCAGCMGCCGCGGTAA	wood	0	1	0	winter	Inoculum wood
10w1		ACTCTGTAATTA	GTGTGYCAGCMGCCGCGGTAA	wood	10	2	61	winter	w10_1
10w2		TCATGGCCTCCG	GTGTGYCAGCMGCCGCGGTAA	wood	10	3	61	winter	w10_2
10w3	806rcbc1443	CAATCATAGGTG	GTGTGYCAGCMGCCGCGGTAA	wood	10	4	61	winter	w10_3
So		GTCAGGTGCGGC	GTGTGYCAGCMGCCGCGGTAA	soil	0	5	0	winter	Inoculum soil
10so1	806rcbc1802	GTTGGACGAAGG	GTGTGYCAGCMGCCGCGGTAA	soil	10	6	61	winter	so10_1
10so2	806rcbc479	GTCACTCCGAAC	GTGTGYCAGCMGCCGCGGTAA	soil	10	7	61	winter	so10_2
10so3	806rcbc561	CGTTCTGGTGGT	GTGTGYCAGCMGCCGCGGTAA	soil	10	8	61	winter	so10_3
Se	806rcbc1234	TTGAACAAGCCA	GTGTGYCAGCMGCCGCGGTAA	sediment	0	9	0	winter	Inoculum sediment
10se1	806rcbc1513	TAGTTCGGTGAC	GTGTGYCAGCMGCCGCGGTAA	sediment	10	10	61	winter	se10_1
10se2	806rcbc1916	TTAATGGATCGG	GTGTGYCAGCMGCCGCGGTAA	sediment	10	11	61	winter	se10_2
10se3	806rcbc1328	TCAAGTCCGCAC	GTGTGYCAGCMGCCGCGGTAA	sediment	10	12	61	winter	se10_3

Caporaso JG, Lauber CL, Walters WA, Berg-Lyons D, Huntley J, Fierer N, Owens SM, Betley J, Fraser L, Bauer M, Gormley N, Gilbert JA, Smith G, Knight R (2012) Ultra-high-throughput microbial community analysis on the Illumina HiSeq and MiSeq platforms. ISME J 6:1621–1624. doi: 10.1038/ismej.2012.8

Table S2

Ternary mixtures	Lignin (%)	Cellulose (%)	Hemicellulose (%)
A	100	0	0
B	0	100	0
C	0	0	100
D	50	25	25
E	25	50	25
F	25	25	50
G	75	25	0
H	25	75	0
I	25	0	75
J	0	25	75
K	33	33	33
L	72	0	25
M	0	75	25

Table S3

	Enzymatic activity						Taxonomy affiliation			
**Strain**	**A**	**B**	**C**	**D**	**E**	**F**	**Closest relative**	**Cover (%)**	**Similarity (%)**	**Accession number**
wB1							*Achromobacter xylosoxidans*	99	99	KT265794
wB2							*Acidovorax soli*	99	99	KT265762
wB3		+		++	+++		*Asticcacaulis benevestitus*	98	98	KT265751
wB4	+	+	+	+			*Chryseobacterium taihuense*	98	99	KT265756
wB5		+		+	+++		*Delftia tsuruhatensis*	99	99	KT265782
wB6	+			+			*Flavobacterium ginsengisoli*	99	99	KT265792
wB7							*Flavobacterium ginsengisoli*	99	99	KT265754
wB8	++		++	+++	+++	++	*Microbacterium gubbeenense*	97	97	KT265752
wB9	+	+	++	++	++		*Microbacterium foliorum*	99	99	KT265781
wB10							*Pseudomonas putida*	97	99	KT265784
wB11							*Pseudomonas putida*	99	99	KT265776
wB12	++	+		++			*Raoultella terrigena*	99	98	KT265749
wB13	++	+		++			*Raoultella terrigena*	97	99	KT265761
wB14							*Sphingobacterium multivorum*	98	98	KT265760
wB15		++	+++	+			*Sphingobacterium multivorum*	99	97	KT265748
wB16		+		+			*Stenotrophomonas terrae*	99	99	KT265788
soB1							*Acinetobacter johnsonii strain*	98	99	KT265766
soB2	++						*Brevundimonas bullata*	95	99	KT265759
soB3	+						*Chryseobacterium taihuense*	99	98	KT265758
soB4				+++			*Citrobacter freundii*	99	99	KT265771
soB5							*Comamonas testosteroni*	96	99	KT265795
soB6							*Comamonas testosteroni*	99	99	KT265789
soB7							*Comamonas testosteroni*	99	99	KT265775
soB8	+						*Flavobacterium ginsengisoli*	100	98	KT265768
soB9				+			*Flavobacterium ginsengisoli*	91	99	KT265787

Table S3. Continuation

soB10	++			++			*Flavobacterium ginsengisoli*	100	99	KT265777
soB11	++						*Flavobacterium banpakuense*	96	99	KT265796
soB12	+++	+++		+			*Lelliottia amnigena*	100	99	KT265765
soB13							*Lelliottia amnigena*	99	99	KT265774
soB14	+++				+		*Microbacterium oxydans*	99	99	KT265770
soB15							*Mycobacterium septicum*	95	99	KT265753
soB16	+	+	+		+		*Ochrobactrum thiophenivorans*	95	99	KT265790
soB17							*Pseudomonas putida*	99	99	KT265767
soB18							*Pseudomonas oryzihabitan*	100	99	KT265793
soB19	++	+		++			*Raoultella terrigena*	96	98	KT265747
soB20	++	+		+			*Raoultella terrigena*	96	98	KT265778
soB21		++	+++	+	++		*Sphingobacterium multivorum*	100	97	KT265757
soB22	+	++	+++	+	++	+	*Sphingobacterium multivorum*	100	98	KT265750
soB23	++	++	+++	+	++		*Sphingobacterium multivorum*	100	98	KT265779
soB24	+++						*Stenotrophomonas rhizophila*	100	99	KT265769
soB25	++						*Stenotrophomonas rhizophila*	100	99	KT265763
seB1							*Acinetobacter beijerinckii*	98	99	KT265764
seB2							*Delftia tsuruhatensis*	96	99	KT265797
seB3	+			+++			*Lelliottia amnigena*	100	99	KT265773
seB4	+	+		+			*Lelliottia amnigena*	100	99	KT265772
seB5	+						*Oerskovia enterophila*	97	99	KT265785
seB6							*Pseudomonas putida*	100	99	KT265786
seB7	+				++		*Pseudomonas salomonii*	97	98	KT265791
seB8							*Pseudomonas putida*	97	99	KT265783
seB9	+	+		+			*Raoultella terrigena*	100	99	KT265755
seB10	+	+	+++	++			*Sphingobacterium faecium*	99	98	KT265798
seB11	+						*Stenotrophomonas rhizophila*	100	99	KT265780

Table S4

Strain	From consortium	Identification (% Identity)*	Activity in glucose	Activity in CMC	Activity in xylan	Activity in cellulose	Accession number
wF1	wood	*Pseudocercospora humuli* (89%)	+	+	+	+	KT265799
wF2	wood	*Arthrographis kalrae* (96%)	+	+	+	+	KT265800
wF3	wood	*Lecythophora sp.* (92%)	++	+	++	+	KT265801
wF4	wood	*Exophiala capensis* (92%)					KT265802
wF5	wood	*Herpotrichiellaceae sp.* (91%)					KT265803
wF6	wood	*Rhodotorula mucilaginosa* (89%)	+	+	+	+	KT265804
soF1	soil	*Acremonium sp*. (97%)	+++	+++	+++	+++	KT265805
soF2	soil	*Mycosphaerella pyri* (90%)	+	+		+	KT265806
soB15	soil	*Mycobacterium septicum* (99%)	+++	+++	+++	+++	KT265753
sedF1	sediment	*Coniochaeta ligniaria* (96%)	+	+	+	+	KT265807
sedF3	sediment	*Penicillium citrinum* (99%)	+++	+++	+++	+++	KT265809
sedF4	sediment	*Plectosphaerella cucumerina* (95%)	+++	+++	+++	+++	KT265810

*Closest relative species –"best" hit partial 18S rRNA gene

EMS 1

Isolation of bacterial and fungal strains. Serial dilutions were done in MSM and 100 µL aliquots of the 10^{-1}- to 10^{-3} and 10^{-7} to 10^{-9} dilutions, for fungi and bacteria, respectively, were spread on the surface of each of the media. Morphological differences of the colonies were used to select the isolates, which were streaked to purity and then preserved at -80°C (in LB broth with 20% glycerol). To obtain a presumptive identification, genomic DNA was produced by using the UltraClean® Microbial DNA isolation kit (MoBio®). For bacteria, we first de-replicated the isolates based on an ERIC-PCR using primers ERIC1R and ERIC2 (Versalovic et al. 1994; Puentes-Téllez and Elsas 2014). The ERIC-PCR cluster analyses were performed using GelCompar software. Bacterial 16S rRNA genes of representative strains for each ERIC group were amplified using 10 ng of DNA and primers B8F and U1406R (Taketani et al. 2010). For fungal strains (pretreated with liquid nitrogen), genomic DNA was obtained using UltraClean® Microbial DNA isolation kit (MoBio®).

Hemicellulosic and cellulosic activities of isolated strains. To assess cellulose degradation capacities, we used 5-bromo-4-chloro-3-indolyl-α-D-glucopyranoside (X-glucopyranoside) and X-cellobiose for detection of α-D-glucosidase and β-D-glucosidase, respectively. For hemicellulose breakdown were tested X-mannopyranoside, X-galactopyranoside, X-xylopyranoside and X-fucopyranoside for detection of α-D-mannosidase, β-D-galactosidase, β-D-xylosidase and α-L-fucosidase, respectively (Sigma-Aldrich, Missouri, USA). Strains were grown on R2A agar Petri dishes in which each compound was spread at a final concentration of 40 µg/mL. The dishes were incubated for 48 h at 28 °C and then stored at 4 °C. The systems were monitored every 12 h. Screenings for (hemi)cellulolytic activity of the fungal isolates were done in mineral medium agar (MMA: 0.2% $NaNO^3$, 0.1% K_2HPO_4, 0.05% $MgSO_4$. 0.05% KCl, 0.08% peptone, 1.5% agar) supplemented with 0.2% glucose, 0.2% CMC, 2% cellulose or 0.2% xylan from beechwood (Fig.S7). Fungal strains were grown on PDA, using agar plugs (5 mm dia) containing grown mycelium that were excised from plates of each isolate and placed in the center of the new agar plate. All assays were performed in duplicate, using as a negative control MMA without an added carbon source. The plates were incubated at 28°C for 7 days, after which they were flooded with Gram iodine. The production of a yellow halo around growth indicated the production of extracellular degradative enzymes (Kasana et al. 2008) for CMC, cellulose and xylan, respectively .

References

Kasana RC, Salwan R, Dhar H, Dutt S, Gulati A (2008) A rapid and easy method for the detection of microbial cellulases on agar plates using gram's iodine. Curr Microbiol 57:503–507. doi: 10.1007/s00284-008-9276-8

Puentes-Téllez PE, van Elsas JD (2014) Sympatric metabolic diversification of experimentally evolved *Escherichia coli* in a complex environment. Antonie Van Leeuwenhoek 106:565–576. doi: 10.1007/s10482-014-0228-y

Taketani RG, Franco NO, Rosado AS, van Elsas JD (2010) Microbial community response to a simulated hydrocarbon spill in mangrove sediments. J Microbiol Seoul Korea 48:7–15. doi: 10.1007/s12275-009-0147-1

Versalovic J, Schneider M, De Bruijn FJ, Lupski JR (1994) Genomic fingerprinting of bacteria using repetitive sequence-based polymerase chain reaction. Methods in Molecular and Cellular Biology 5:25–40.

Fig. S1

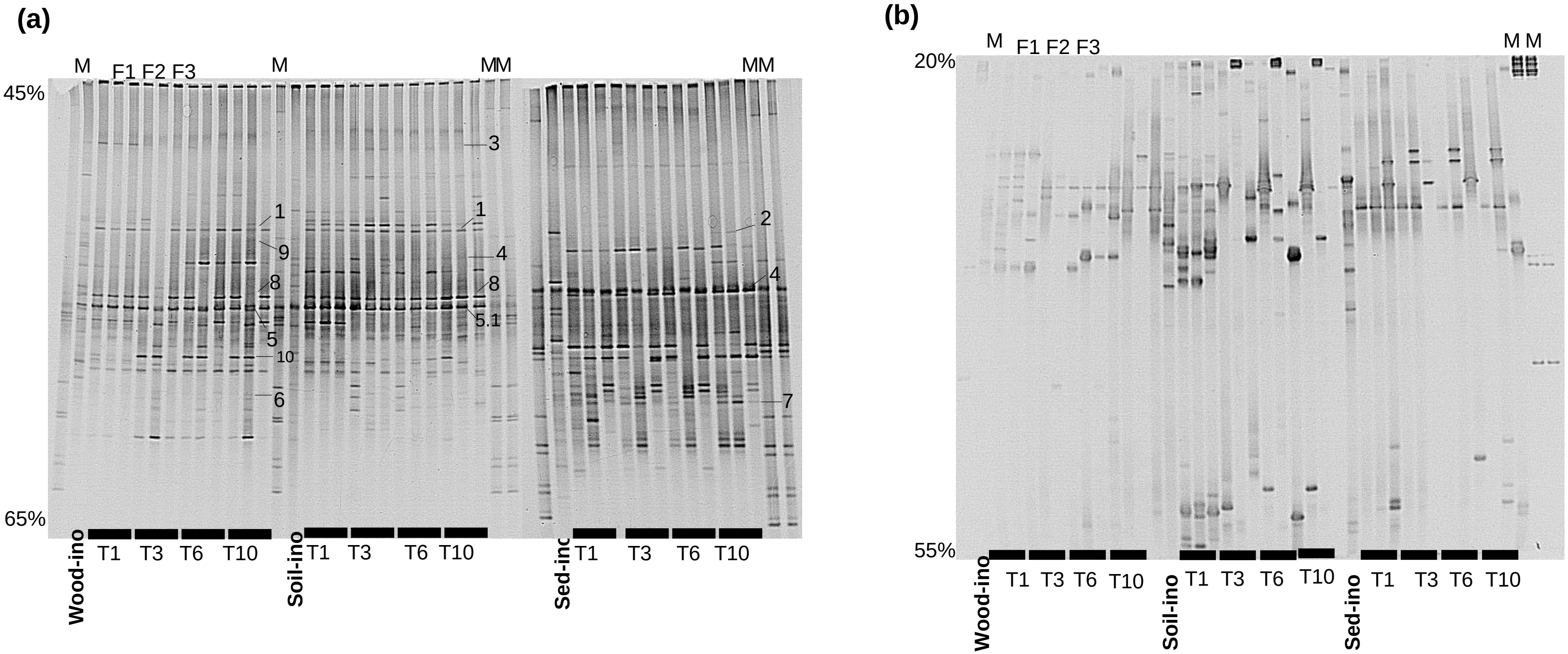

Fig. S1 Analyses of steps of the enrichment process. PCR-DGGE analyses of **a)** bacterial and **b)** fungal communities at different transfer steps (T1, T3, T6 and T10). The DGGE patterns showed a reduction in the number of bands over experimental time for each of the three inocula. Several bacteria DGGE bands were presumptively identified as being derived from several strains **(a)**. The matching was as follows: *Sphingobacterium multivorum* (1), *S. faecium* (2), *Citrobacter freundii* (3), *Lelliottia amnigena* (4), *Acinetobacter johnsonii* (5), *A. beijerinckii* (5.1), *Pseudomonas putida*(6), *P. salomonii* (7), *Flavobacterium ginsengisoli* (8), *Chryseobacterium taihuense* (9), *Asticcacaulis benevestitus* (10). M: marker

Fig. S2

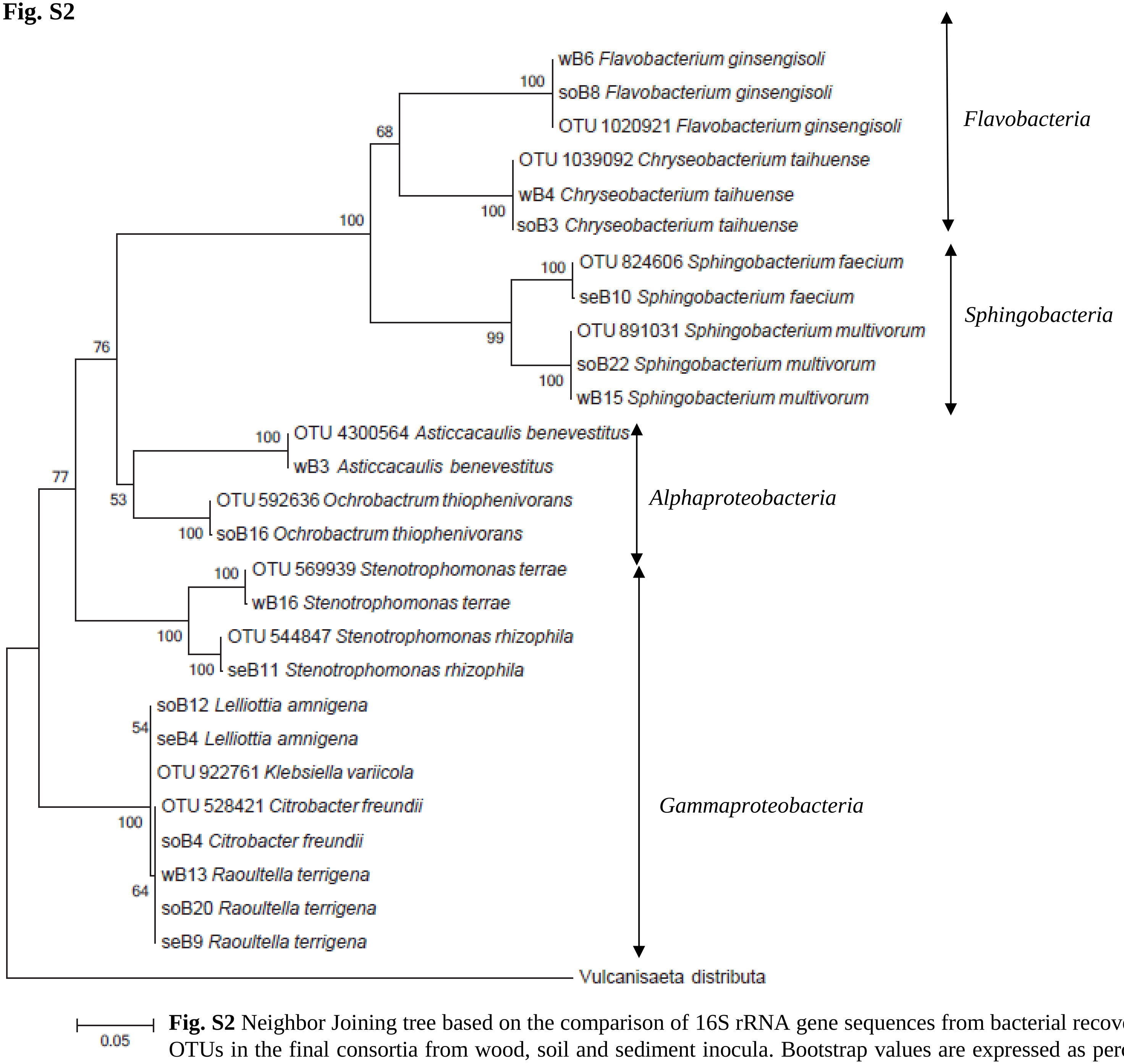

Fig. S2 Neighbor Joining tree based on the comparison of 16S rRNA gene sequences from bacterial recovered strains and the most abundant OTUs in the final consortia from wood, soil and sediment inocula. Bootstrap values are expressed as percentages of 1000 replications. The scale bar estimates the number of substitutions per site. The name in the right part correspond to the taxonomic Class.

Fig. S3

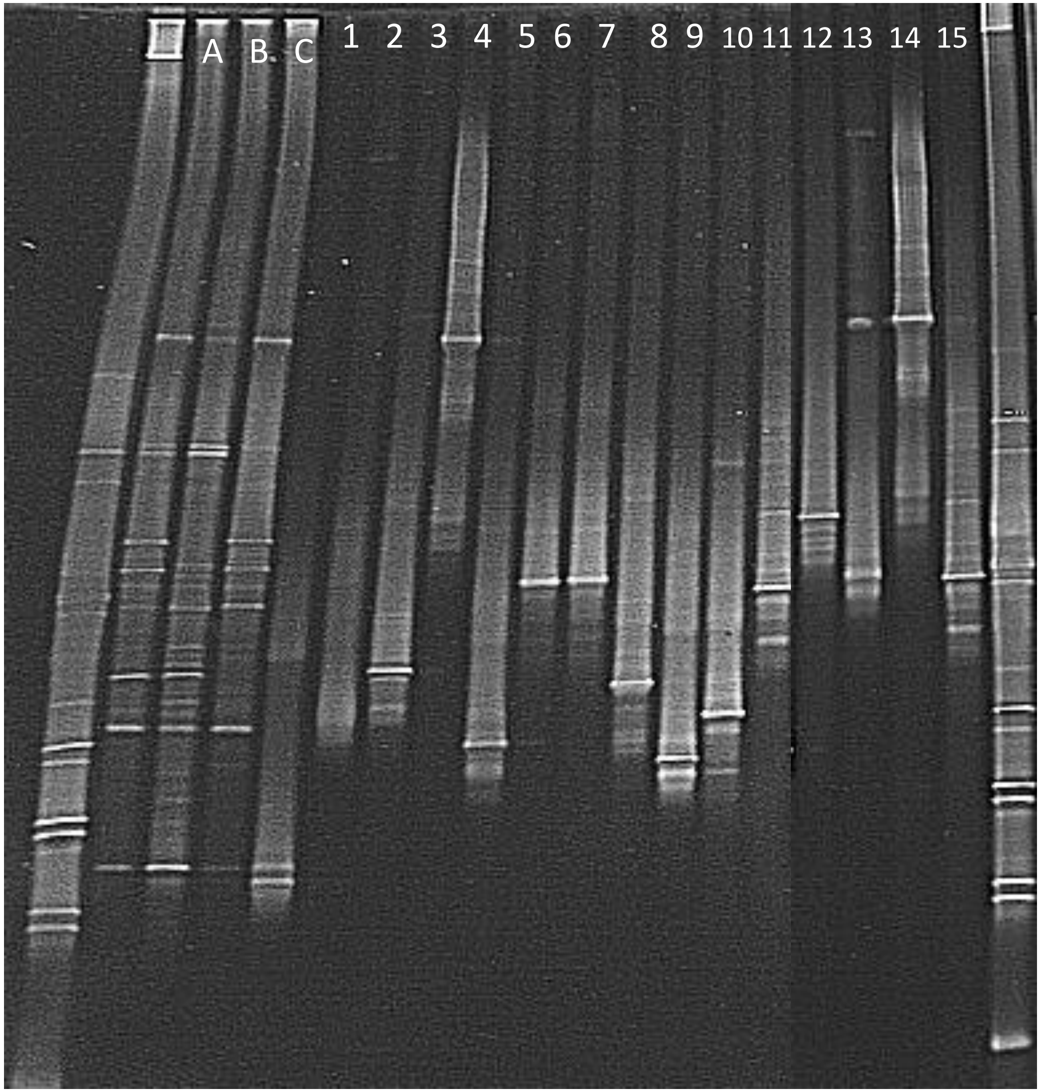

Line	Sample
A	Wood derived consortia (T10), flask 1
B	Wood derived consortia (T10), flask 2
C	Wood derived consortia (T10), flask 3
1	*Achromobacter xylosoxidans*
2	*Acidovorax soli*
3	*Asticcacaulis benevestitus*
4	*Chryseobacterium taihuense*
5	*Delftia tsuruhatensis*
6	*Flavobacterium ginsengisoli*
7	*Flavobacterium ginsengisoli*
8	*Microbacterium gubbeenense*
9	*Microbacterium foliorum*
10	*Pseudomonas putida*
11	*Stenotrophomonas terrae*
12	*Raoultella terrigena*
13	*Sphingobacterium multivorum*
14	*Sphingobacterium multivorum*
15	*Stenotrophomonas terrae*

Fig. S3 Co-migration DGGE of enriched wood derived consortia community (T10) and recovered bacteria strains

Fig. S4

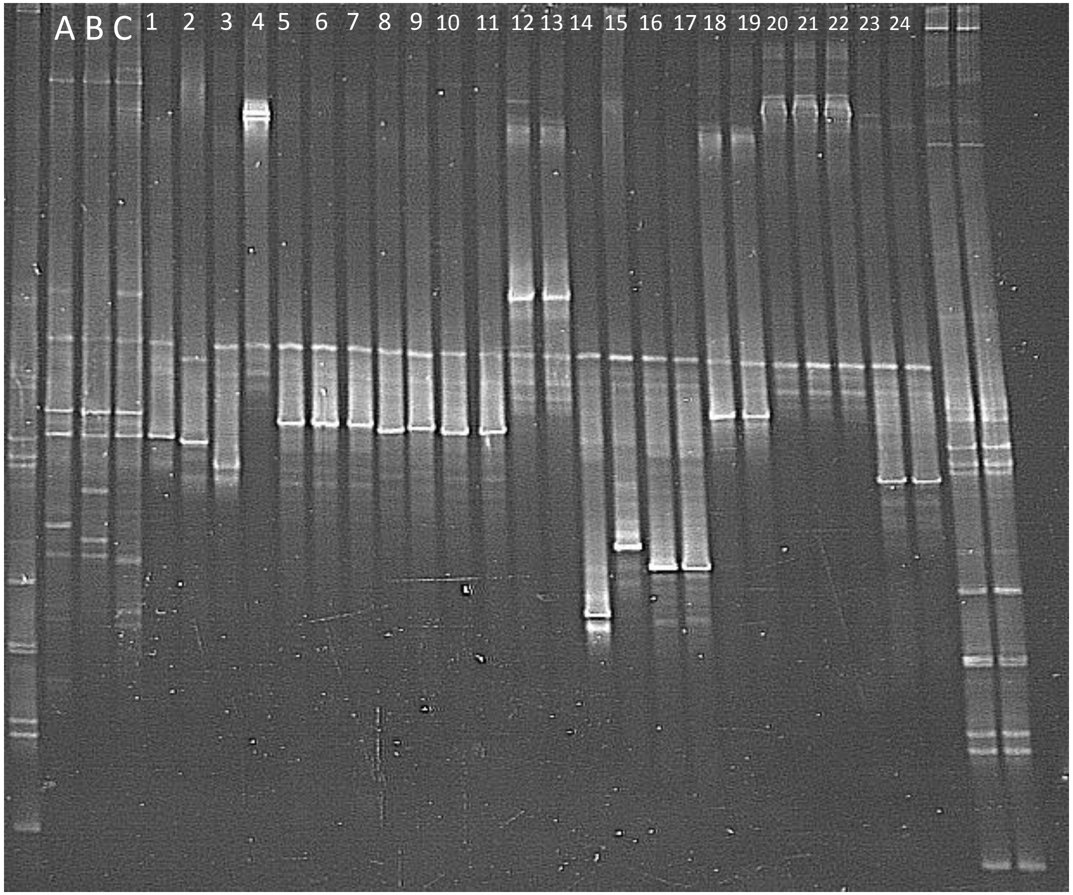

Line	Sample
A	Soil derived consortia transfer (T10), flask 1
B	Soil derived consortia (T10), flask 2
C	Soil derived consortia (T10), flask 3
1	*Acinetobacter johnsonii*
2	*Brevundimonas bullata*
3	*Chryseobacterium taihuense*
4	*Citrobacter freundii*
5	*Comamonas testosteroni*
6	*Comamonas testosteroni*
7	*Comamonas testosteroni*
8	*Flavobacterium banpakuense*
9	*Flavobacterium ginsengisoli*
10	*Flavobacterium ginsengisoli*
11	*Flavobacterium ginsengisoli*
12	*Lelliottia amnigena*
13	*Lelliottia amnigena*
14	*Microbacterium oxydans*
15	*Ochrobactrum thiophenivorans*
16	*Pseudomonas oryzihabitans*
17	*Pseudomonas putida*
18	*Raoultella terrigena*
19	*Raoultella terrigena*
20	*Sphingobacterium multivorum*
21	*Sphingobacterium multivorum*
22	*Sphingobacterium multivorum*
23	*Stenotrophomonas rhizophila*
24	*Stenotrophomonas rhizophila*

Fig. S4 Co-migration DGGE analysis of enriched soil derived consortia community (T10) and recovered bacteria strains

Fig. S5

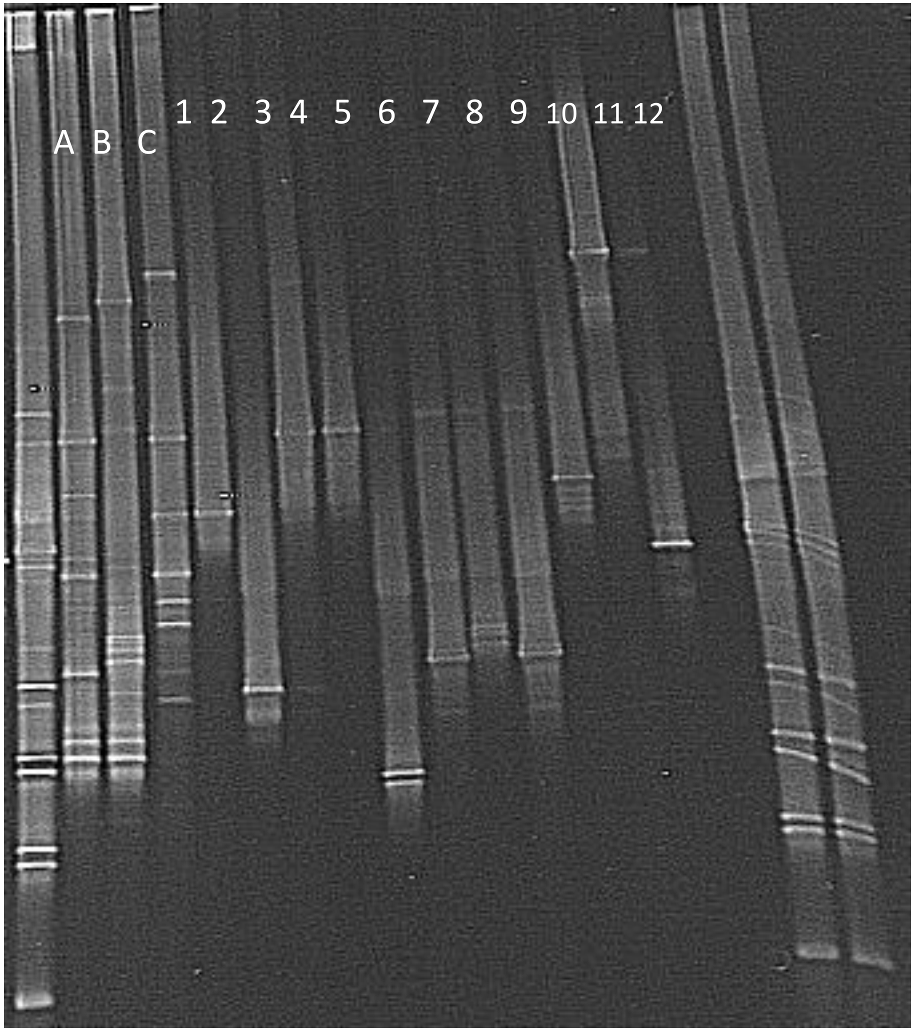

Line	Sample
A	Sediment derived consortia (T10), flask 1
B	Sediment derived consortia (T10), flask 2
C	Sediment derived consortia (T10), flask 3
1	*Acinetobacter beijerinckii*
2	*Delftia tsuruhatensis*
3	*Lelliottia amnigena*
4	*Lelliottia amnigena*
5	*Oerskovia enterophila*
6	*Pseudomonas putida*
7	*Pseudomonas salomonii*
8	*Pseudomonas putida*
9	*Raoultella terrigena*
10	*Sphingobacterium faecium*
11	*Stenotrophomonas rhizophila*
12	Negative control

Fig. S5 Co-migration DGGE analysis of enriched sediment derived consortia community (T10) and recovered bacteria strains

Fig. S6

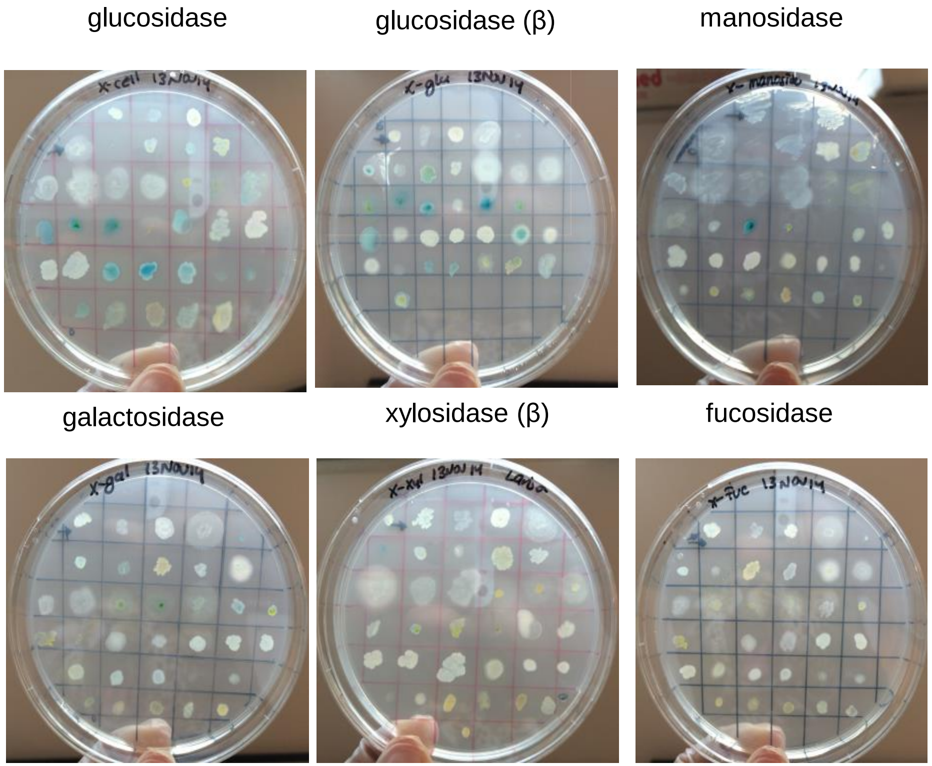

Fig. S6 Enzymatic activity detection by chromogenic substrate, in active bacterial strains isolated from final wood, soil and sediment derived consortia.

Fig. S7

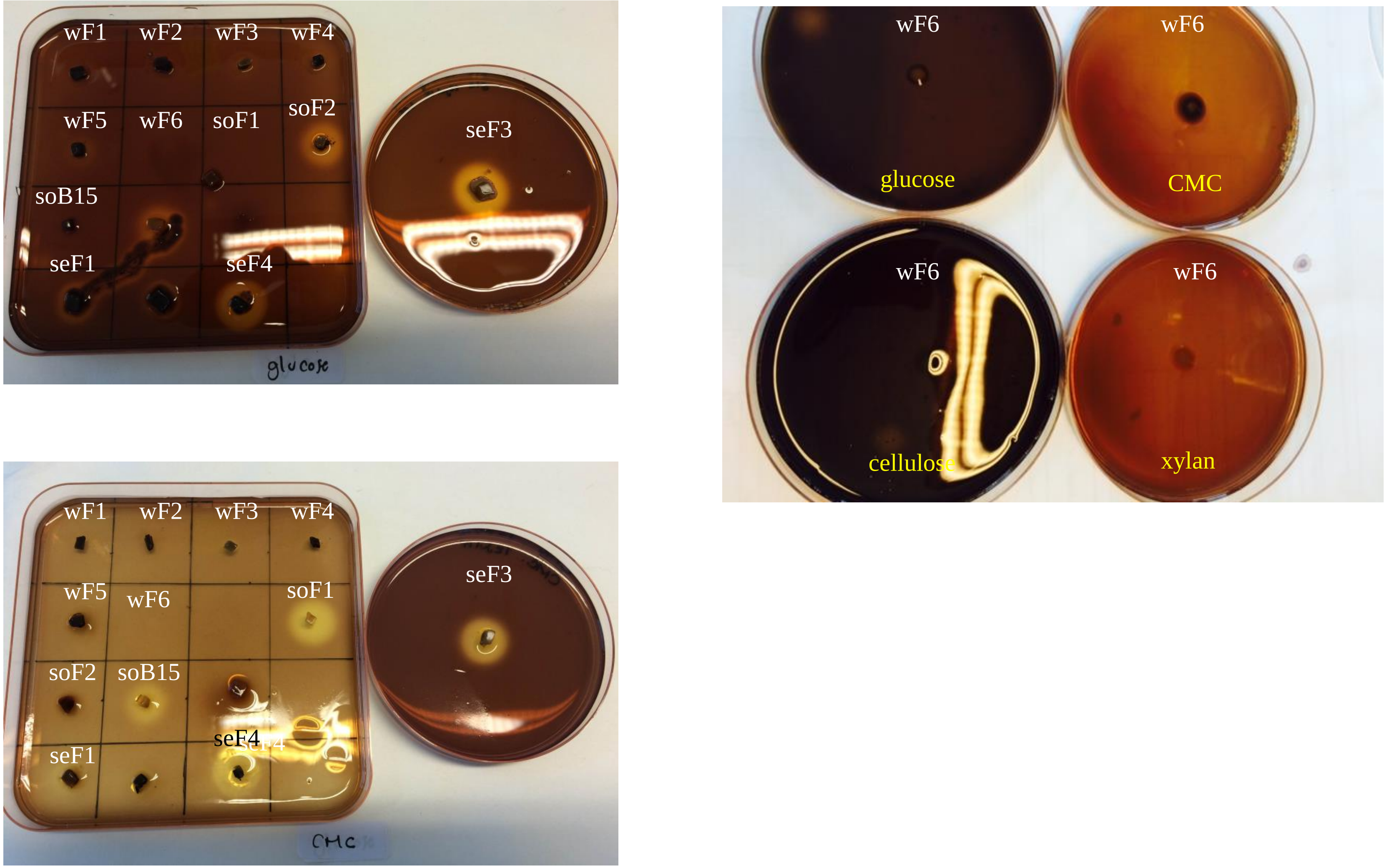

Fig. S7 Enzymatic activity detection in CMC, xylan, and cellulose of fungal isolated from final wood, soil and sediment consortia
